# Intellectual capital-based performance improvement: a study in healthcare sector

**DOI:** 10.1186/s12913-021-06087-y

**Published:** 2021-01-20

**Authors:** Simona Alfiero, Valerio Brescia, Fabrizio Bert

**Affiliations:** 1 Department of Management, C.so Unione Sovietica, University of Turin, 218 bis, Torino, Italy; 2 Department of Public Health Sciences, University of Turin, Via Santena 5/bis, Torino, Italy

**Keywords:** Performance, Intellectual capital, Efficiency, Healthcare sector, Data envelopment analysis

## Abstract

**Background:**

Knowledge resources are in most productive sectors distinctive in terms of competitiveness. Still, in the health sector, they can have an impact on the health of the population, help make the organisations more efficient and can help improve decision-making processes. The purpose of this paper is to investigate the Intellectual Capital impact on healthcare organization’ performance in the Italian healthcare system.

**Methods:**

The theoretical framework linked to intellectual Capital in the health sector and the performance evaluation related to efficiency supports the analysis carried out in two stages to determine the right placement of resources and the exogenous variables that influence performance level. The evaluation of the impact of the ICs on performance is determined through the Data envelopment analysis. The incidence of the exogenous variables has been established through linear regression.

**Results:**

Empirical results in Italy show some IC components influence organization ‘performance (Essential Levels of Assistance) and could be used for defining the policy of allocation of resources in healthcare sector. The efficiency of 16 regions considered in 2016 based on Slack-Based-Model constant returns-to-scale (SBM-CRS) and Slack-Based-Model variable returns-to-scale (SBM-VRS) identifies a different ability to balance IC and performance. Current healthcare expenditure and the number of residents is correlated with the identified efficiency and performance levels.

**Conclusions:**

This paper embeds an innovative link between healthcare performance, in term of efficiency and IC which aligns resource management with future strategy. The study provides a new decision-making approach.

## Background

Currently, the search for efficiency and effectiveness conducted through the introduction of management tools as Performance Measurement System (PMS) by New Public Management (NPM) has had several positive results, especially in Western countries [1]. However, in the public and healthcare sector, change is tied to governance tools capable of pushing towards a more sustainable approach [2] and to the increasing importance of knowledge as the key factor to achieve competitive advantage. The literature shows that healthcare organizations are conditioned by knowledge-intensive and austerity policies that push for the identification of methods to efficiently reduce and manage the funds and resources granted to the healthcare sector [3, 4].

The objective of the twenty-first century, in fact, promoted among the objectives of Europe 2020 is to manage the knowledge and the intellectual capital (IC) within organizations to maximize efficiency by identifying innovative solutions to reduce resource consumption and lead to a structural, organizational and process change [5, 6]. In particular, the literature highlights an increase in the demand for performance measurement to increase the quality of services and the sustainability of healthcare organizations [3, 7]. The analysis of the organization and, in particular, of the IC’s elements can be considered essential in the decision-making process within health organizations [3, 8]. Although the intangible asset and many elements that make up the IC are not representative in the financial statement [9], they are essential organizational elements to create an organizational advantage [10]. In the private sector, many studies confirm that the IC significantly affects performance [11, 12] and competitiveness [13–16],but in the health sector, research on intellectual capital management from an empirical perspective still seems to be insufficient [17]. How to leverage intellectual capital effectively and its impacts on performance has seldom been investigated empirically [18, 19] and is still subject to further study. The literature highlights fews studies in the health sector at the regional and national levels that stress the relationship between performance and IC and, in particular, the balance between the kinds of resources [3, 17, 20, 21]. Empirical studies on the European context are not yet numerous, and there are no studies that apply the Data envelopment analysis to the health context [17]. The Italian context’s particular characteristics reveal the need for a new set of techniques capable of managing ICs efficiently [22]. The measurement often represents the relationship between IC and performance in health through Balance Scorecard, which pays attention to both financial and non-financial indicators simultaneously; the two groups often find an overlap that does not adequately identify inputs and outputs, limiting the real measurement of impact and outcomes [21]. The study aims to identify an effective method of measuring ICs equilibrium.

The research aims to contribute to intellectual capital in the healthcare sector by integrating the gap identified in the literature through the empirical analysis of the influence of intellectual capital on performance on the regional healthcare system through two investigation stages. The first intends to evaluate the impact of intellectual capital on performance and the second stage that wants to consider exogenous elements that affect performance.

In the first stage, the research uses the data envelopment analysis, which is a typical performance assessment approach to evaluate the efficiency of organizations. Data envelopment analysis is particularly useful for practitioners to adopt benchmarking, as entities can quickly identify the efforts required to catch up with benchmarking partners by examining their performances. In the past, many publications have adopted Data envelopment analysis as evaluation techniques to assess the efficiency; however, to date, there is no application on intellectual capital management performance in healthcare. In the analysis we focused our attention precisely on the relationship between input and output, or rather on the effects of the Intellectual Capital elements on performance [23]. In the second stage, through a regression, we analyse how exogenous factors affect the efficiency score achieved.

The study focuses on the Italian regions and its health system. The Italian state is considered as an example of the first countries in the world according to the Bloomberg Health-Care Efficiency classification which calculates based on World Bank, WHO, United Nations and IMF data which are the most efficient health systems analyzing the relationship between costs and expectation of life [24]. The Italian national health system is public and universal and organized by the national and regional levels. The Ministry of Health, through some instrumental bodies, maps the achievement of Essential Assistance Levels in conditions of appropriateness and efficiency in the use of resources, as well as the congruity between the services to be provided and the resources made available. The Essential Levels of Assistance defined through a single parameterized indicator on the assistance activity in living and working environments, territorial assistance, and hospital assistance identify in our study an element of evaluation of the non-financial performance achieved [25, 26]. The balance of ICs considered as a function of the non-financial parameter seeks through a second analysis the possible relationship to a financial parameter linked to the expenditure of Italian companies.

This study has the following objectives: (1) to establish an assessment model to measure the intellectual capital of Italian regions and its health system and use this model to identify the regions that are on the efficient frontier; (2) to evaluate the performance of 21 Italian regions and its health systems; (3) to verify the amount of slack for inefficient regions to improve; (4) to highlight the relationship between exogenous elements and performance in the healthcare sector.

The paper is structured as follows: in Section 2, theoretical literature is reviewed in relation to IC in healthcare sector and performance evaluation; in Section 3, the research framework is explained and two applied methods, Data envelopment analysis and linear regression, are briefly discussed. The results of empirical study are articulated in Section 4; followed by the discussion of findings and research conclusions in the last sections.

### Literature review

The analysis of the literature carried out highlights the relationship between the definition of IC and health care performance. The next paragraph is dedicated to the analysis of the literature on IC in the healthcare sector up to current studies, and finally healthcare performance evaluation is analyzed in depth.

### Intellectual capital in healthcare sector and current studies

The intellectual capital can be viewed as the complex organization process that incorporates knowledge management, “best practice” transfer and organizational learning and can turn employees’ skills, knowledge and expertise into values that are vital to organizational performance [27]. Guthrie et al. [28] describe the development of to the IC literature of the past two decades. The first period from 1980 to 1990 was based on the IC concept’s development and on the importance to create competitive advantages. The second stage is based on approaches to measure the ICs and define their various components. The latest period, started from 2000, highlights at least 50 methods by which ICs are managed [29]. The study conducted takes place in the last period and identify variables of the Italian national health system using methodology already applied in other contexts. Some authors linked the IC to three elements of the triple bottom line related to the concept of sustainability or rather human capital, relational capital and structural capital [20, 30]. The study by Cavicchi & Vagnoni [20] highlights the need, through a questionnaire, to investigate the sustainability and impact that ICs have on the health system, inviting the identification of new methodologies to measure its balance.

Although there is no common definition of intellectual capital [31, 32] the approach based on the three elements is the most widespread and constitutes the current framework of reference. A study conducted by Pedro and Alves [17] justifies what has been identified by the study of the existing literature, with which we have integrated what has already been identified in the taxonomy relating to Intellectual capital, focusing it on the health sector.

Human capital represents in the organization a series of elements that refer to the knowledge and skills of workers [16, 33]. Human capital can be identified within organizations as the attitude and motivation of workers, skills, abilities, creativity and innovation, experience, personal characteristics, knowledge and efficiency [10, 15, 20, 34–40]. These elements depend on the type of sector and company to which they refer [41]. The main drivers in healthcare organizations related to human capital are skills and knowledge [21].

Relational capital is defined as the value of the organization’s brand, strong relationship with customer, consumer satisfaction [15, 19, 30, 31, 42, 43]. Relational capital therefore refers to the capital knowledge generated by the relationship with external stakeholders [44, 45]. The quality of relationships and the ability to increase customers are the key elements within any organization [46]. In healthcare, the relationship between doctor and healthcare worker and patient is the basis of the care relationship and affects the quality of the output, the element also allows to understand the communication capacity and the exchange of knowledge that also leads to the personalization of the care and to a greater effectiveness [47]. Relational capital in healthcare is also based on the type of network that the organization manages to build with its stakeholders, including users, universities and governments [11, 21, 48].

Structural capital is defined as a set of technologies, inventions, data, publications, strategies, culture, structure and system, a set of activities and procedures that the organization brings together [21, 31, 35, 41, 49, 50]. Structural capital can also be defined as the set of organizational properties that affect both the process and the creation of innovative capital [12, 41, 51]. The technology adopted in the various structures plays an important part in healthcare compared to the others, which can be difficult to standardize [20, 21].

Numerous studies have dealt with IC within the health system. Some papers investigate the meaning of IC through literature to identify a common language and highlight the absence of empirical studies that justify the use of IC at the regional and national levels [17, 21]. The study conducted on the Taiwan case through questionnaires addressed to hospital managers highlights how IC and performance measurements are correlated and essential in the management of hospital policies. In particular, human capital is the most critical IC, and staff costs are the most representative performance indicator. However, these are parameters referring to the local context that provides a priority in the organizational capital rather than a capacity for formulating future strategies [19]. Simultaneously, semi-structured interviews conducted in Norway, UK, and Germany focused on the relationship between performance and IC highlights that healthcare managers already consider IC, and that new measurement tools suitable for the context of reference are needed [22]. Both studies aim at the personalization of the tools concerning the local context. The application identified in the study by Pirozzi & Ferulano [3] adapt and propose a new framework for the analysis of ICs and performance starting from the model of Emilia Romagna (Italy) and the English health system, without however implementing it with empirical evidence: The same study directs the search for new theoretical frameworks suitable for the context. The questionnaire is the most used method to identify relationships and implications between the management of ICs and the organizational and financial fallout, for example, 277 respondents allowed hospitals in Taiwan to identify a relationship between the variables of human capital and the economic growth of the healthcare sector [16]. The adoption of a questionnaire is also identified in the study by Cheng et al. which uses the research tool to confirm the relationship between input (financial resources and IC) and output (performance identified in the customer relationship). That survey invites us to study the impact on economic performance and the impact on performance in the future finance hospitals [52]. The study by Vishnu and Vijay [53] helps the researchers retrace the studies previously conducted on the subject of IC and impact on performance, identifying numerous applications in Asian countries, and only one in western ones. In all cases, the questionnaire is the commonly applied method that definitively confirms the relationship with the organizational and financial health performance according to the perception of the professionals, without however giving explicit evidence [17, 21]. Therefore, the models provided are rarely confirmed empirically due to a rare collection and uniformity of the variables adopted.

### Healthcare performance evaluation

Since 1980, drastic changes have been adopted through New Public Management (NPM) that have introduced privatized principles and instruments [54, 55]. The new assumption of western countries became “lean and more competitive while, at the same time, trying to make public administration more responsible to citizen’s need by offering value for money, choice, flexibility, and transparency” (OECD, 1993). Performance measurement must be considered from a political point of view especially in a period of austerity. Bouckaert et al. highlight how resources can be studied as a black box, where in front of a set of resources provided by the government, services are offered that lead to outcomes that guide decisions within the managerial and political cycle [3]. Aging and recession led to the search for effective sustainability of resources, which already in 2017 had absorbed 20% of the GDP in many OECD countries, pushing academics and politicians to search for managerial and technological solutions [56]. The literature frequently identifies the Balanced scorecard as the best method of evaluating financial and non-financial performance [57]. However, several studies identify the method as backward and unable to represent the real relationship between IC and performance in defining desired outcomes [3, 17, 21]. The use of Performance Measurement System (PMS) was recommended to facilitate the implementation of strategies and organizational performance [3, 52]. PMS takes into consideration a series of financial and non-financial results, if the financial results were introduced immediately with the introduction of the NPM, the non-financial results were considered only as a result of the difference in value between the market value and the balance sheet given right from intellectual capital [3, 16, 52]. The introduction of new management tool capable of taking performance into account is useful for a better definition of needs, and resource allocation has been made necessary by the reduction of resources in reference to an increase in the aging of the population with an impact on performance outcomes [58–61]. According to the data of World Population Prospects (2017), the number of older persons those aged 60 years or over is expected to more than double by 2050 and to more than triple by 2100, rising from 962 million globally in 2017 to 2.1 billion in 2050 and 3.1 billion in 2100. Globally, population aged 60 or over is growing faster than all younger age groups. The increase in population will undoubtedly lead to the need to place the available resources appropriately, do not impact negatively on the performance [62, 63]. The various elements that are not present very often in the financial statements relating to the IC must be considered to obtain better performance levels. According to the most common approach, the performance and output of the health service are measured as percentages of mortality by performance [64]. This indicator does not allow a real assessment of reality, which can be instead expressed by a multitude of indicators, synthesized by access, accessibility, applicability, environmental and services care, proficiency, Effectiveness or attention to health or clinical awareness, expense or cost, Effectiveness, equity, governance, centrality of patient, attention to responsibility, safety, sustainability, and rapidity [65, 66]. Each system then implements a mix of these factors [67]. The Italian system uses most of these criteria to evaluate performance through a system of compound indicators called Essential Levels of Assistance (LEA) [64].

Essential Levels of Assistance can also be defined as the level of health service standards capable of mapping 21 health activities through enhancements standardized related to performance and its achievement [68, 69]. The State-Regions agreement of 23 March 2005 entrusts the Verification of Fulfilments, which the regions are required to, to the Standing Committee for the verification of the delivery of the Essential Levels of Assistance in conditions of appropriateness and effectiveness in the use of resources (briefly renamed as the Essential Levels of Assistance Committee) which together with the Compliance Check Table, allows the regions involved. The assessment of the actual quality provided is verified by the Ministry of Health Italy, by the Italian Medicines Agency and by the National Agency for Regional Health Services, competent in the matters of compliance, and subsequently examined and validated by the members of the Essential Levels of Assistance Committee. The certification of the fulfilment relating to the “maintenance in the provision of the Essential Levels of Assistance “ takes place through the use of a defined set of indicators divided between the assistance activity in the living and working environments, the district assistance and the hospital care, collected in a grid (so-called LEA grid) which allows to know and understand the diversity and the uneven level of delivery of the levels of care. A score above 160 is considered positive, not detecting critical indicators in terms of performance and output. The analysis conducted focuses on regional IC elements and performance in Italy. The study is useful to redefine the healthcare policy based on IC, but for a global application it will have to consider the particularities and differences of each state [70–72].

## Method

### First stage

The research is mainly based on the IC elements highlighted by the literature and who they impact on Essential Levels of Assistance.

Data envelopment analysis was proposed by Charnes et al. [73], and it is a linear programming technique which can be used to determine the efficiency of a group of decision-making units (DMUs) relative to an envelope (efficient frontier) by optimally weighting inputs and outputs. Additionally, data envelopment analysis provides a single indicator of efficiency irrespective of the number of inputs and outputs. Data envelopment analysis has been applied in a number of fields, including education institution [74], healthcare [75–83], banking [84, 85], manufacturing [86, 87], food sector [88–91].

While data envelopment analysis is now widely recognized as an evaluation approach for performance analysis of various DMUs, to date, there is no direct application on intellectual capital management performance in the healthcare sector. The choice to adopt this technique is represented by the fact that its operation is conditioned by the size of the sample, which in the empirical analysis leads to a higher sensitivity linked to the obtainable result [92–95].

The set of DMU performances that represent best practices are assigned based on efficiency levels. Therefore, the technique after establishing an efficient frontier assigns the distance from these levels by assigning an efficiency value. Data envelopment analysis uses several types of models, but they can be largely classified into a constant returns-to-scale (CRS) model and a variable returns-to-scale (VRS) model, depending on size variability. The CRS model is based on the assumption that the input and output ratios do not change with size and it is an estimation of overall technical efficiency (OTE). In data envelopment analysis, OTE measure has been broken down into two mutually exclusive and non-additive components: pure technical efficiency (PTE) and scale efficiency (SE). This break down provides an insight into the source of inefficiencies. The PTE measure is obtained by estimating the efficient frontier under the assumption of variable returns-to-scale. It is a measure of technical efficiency without scale efficiency and only reflects the managerial performance to organize the inputs in the production process [96], and it has been used as an index to capture managerial performance. The ratio of OTE to PTE provides SE measure. The measure of SE provides the ability of the management to choose the optimum size. The VRS model applies when the ratio of input and output varies in size; it is also called the BCC model after Banker et al. [97], whos first introduced it.

The VRS model searches for PTE (also called managerial efficiency) and includes the so-called convexity constraints by changing the specification of the problem and providing the measure of Managerial Efficiency θ VRS, adding eλ = 1 to the programme (θ is a scalar and λ is a vector of constants). From its inception, the data envelopment analysis has treated each DMU as a “black box” by only considering those inputs consumed and the final outputs produced by this “black box” [98]. However, the efficiency measure is associated with the use of a minimum number of inputs in order to produce a certain number of outputs or the maximum production of outputs using a certain number of inputs [99] from which the orientation issues descend.

There are two types of data envelopment analysis models: the radial (CCR) and the non-radial. CCR does not consider slacks, which are relevant for evaluating managerial efficiency and reporting the efficiency score. Therefore, the data envelopment analysis frameworks used in this study are based on the Slack-Based Model (SBM), which is a non-radial model developed by Tone [100]. This model deals with the lack of inputs and outputs of each DMU, called “slacks”, and projecting each DMU to the furthest point on the efficiency frontier by minimizing the objective function and finding the maximum slacks.

The study carry out the output-oriented model, which projects the DMUs on the efficient frontier keeps the inputs level constant by trying to increase outputs proportionally in order to reach the efficient frontier. In order to illustrate the model, let us assume that there are n DMUs (DMUj, j = 1, 2, …, n) with m inputs (xij, i =1, 2, …, m) and s outputs (yrj, r =1, 2, …, s) for each DMU. The ui and vj are the weights corresponding to the ith input and jth output. Then the SBM-DEA model can be described as follows.

$$ \frac{1}{p_o^{\ast }}={\mathit{\max}}_{\lambda, {s}^{-},{s}^{+}}\ 1+\frac{1}{s}\sum \limits_{r=1}^s\frac{s_r^{+}}{y_{ro}} $$

subject to:

$$ \sum \limits_{j=1}^n{\lambda}_{jk}{x}_{ij}+{s}_{ik}^{-}={x}_{ik},\forall i $$

$$ \sum \limits_{j=1}^n{\lambda}_{jk}{y}_{rj}-{s}_{rk}^{+}={y}_{rk},\forall r $$

$$ {\lambda}_{jk}\ge 0,{s}_{ik}^{-}\ge 0,{s}_{rk}^{+}\ge 0,\forall i,r,j,k $$

If the optimal value ${\lambda}_{jk}^{\ast }$ of *λ*_*jk*_ is non-zero, then the jth region represents the reference set (peers) for the kth region, and the corresponding optimal value is known as the peer weight of the jth region.

The numerator value evaluates the mean of inputs. Similarly, the reciprocal of the denominator evaluates the mean expansion rate of outputs. This model is known as SBM-CRS model [100].

The kth region is said to be Pareto efficient if all slacks are 0, i.e., ${s}_{ik}^{-\ast }={s}_{rk}^{+\ast }=0$ for all i and r, which is equivalent to ${p}_k^{\ast }$ = 1. The non-zero slacks and (or) ${p}_k^{\ast }$ < 1 identify the sources and amount of any inefficiency. The reference set showed how input and output can be increased to be the Kth Region. Subsequently, the PTE is determined by the SMB-VRS model and we can calculate SE for every region. However, the results of output-oriented SBM-CRS and SBM-VRS models are calculated using data envelopment analysis Solver.

### Input and output variables

The selection and the number of inputs and outputs are fundamental steps to guarantee a discriminatory power in the data envelopment analysis model. Given the low number of DMUs [16] it is possible to respect the computational requirements to get good discriminatory power with 4 inputs and 1 output [101–104].

In the analysis carried out, the inputs are the elements of IC, while output considers the performance. The study considers the following inputs deemed significant: diagnostic technologies in each region defined through the number of equipment acquired and found as the primary element of the structural capital, medical and nursing staff as a representative element of human capital within the structures [22], continuing education in medicine as an element of representation of the intellectual capital which defines and modifies the corporate culture and affects the ability and skills of each individual and work team and patient satisfaction with regard to the health services received in the care process as a primary element of relation capital in the health sector. For human capital we have chosen to analyse two fundamental dimensions: staffing [22] and training and development [105–107]., that represent the key inputs for an effectiveness human resource management practices [108–110]. Hospital facilities, in fact, are highly knowledge-intensive [20]; several studies highlight the importance of the human factor and training as essential elements for better financial and non-financial performance [3, 22, 106, 111].

The use of technologies, understood as diagnostic tools present within the healthcare facilities, is the selected variable of structural capital, this have also an impact on the performance on the skills of human personnel directly engaged and in contact with the user [17, 105]. The technologies considered include linear accelerator, hemodialysis device, computerized gamma camera, integrated CT gamma camera system, mammography unit, positron emission tomograph, integrated CT / PET system, computerized axial homograph, magnetic resonance tomograph. Customer satisfaction is one of the main elements in defining the relationship between patient and organization, very often it is conditioned by other intellectual capital such as personnel, technology, protocols and response time but it is the capital that best represents the ability of operators to relate and respond to the organizational and external need, for this reason, it was selected as relational capital [112].

All input data are provided by the Italian Ministry of Health and collected uniformly at the national level in 2020, ensuring a systematic methodological approach. The 2016 is the last available year and therefore is the one used in the study.

The output of performance can be considered through the LEAs (Essential Levels of Assistance). The set of services to be guaranteed by the public sector is defined at national level, while regions are accountable for their provision. Italian National Health Service is based on this mapping and performance definition criterion [113]. The composite indicator was created by the working group of the Ministry of Health supported by the Italian technical bodies and defines the impact in terms of health performance [26].

**Fig. 1:**
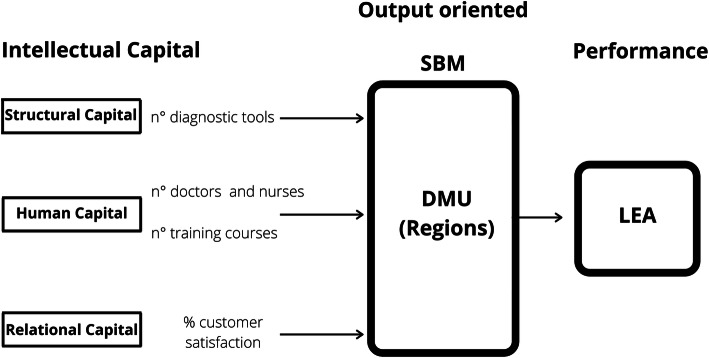
Output oriented SBM. Source of figure: own production

The Fig. 1 and Table 1 show the DEA model and the variables selected.

**Table 1:** Input and output description

Variables	Average	Standard deviation	Min	Max	Input (I)/output (O)
Structural Capital (n° diagnostic tools)	8575.938	6459.741	1103	25399	I
Human capital (n° doctors and nurses)	20,172.5	13,007.67	1753	47247	I
Human capital (knowledge and skills of workers - n° training courses)	1896.5	1506.373	107	5175	I
Relational Capital (consumer satisfaction)	37.761	13.620	19.365	57.535	I
Performance (LEA)	182.437	24.762	124	209	O

Source: Statistical analysis based on [133, 134]

### Second stage

The analysis also identifies elements able to define how the score attributed to each region has a functional relationship with exogenous elements to the IC that could significantly affect the data envelopment analysis analysis conducted.

To understand how exogenous variables, impact on the efficiency level achieved by each DMU, ​​we performed a regression. The number of residents and health expenditure are the elements considered in the analysis as exogenous factors that could influence the ranking of each region in terms of performance according to the literature [114, 115]. Although usually, the increase in public expenditure per capita leads to an improvement in performance, it is still possible to identify conflicting opinions in the literature, which therefore require more investigations [116, 117]. Furthermore, although the number of inhabitants requires higher IC elements as size of structure and human resources [118], there are not many studies that go to analyze the possible relationship, indeed very often, the investigations focus on specific diagnostic and treatment activities [119–121] without carrying out macro-investigations on the systemic incidence. The relationship between the score attributed to each region based on ICs and Essential Levels of Assistance and number of residents and expenditures was verified through multivariate regression and Tobit analysis to confirm the robustness of statistical evidence [122–124]. All statistical analyses were performed using STATA V.13 (Stata Corp, College Station, Texas, USA, 2013) and *p*-value < 0.05 was considered significant for all analyses.

### Sample and description

The study considers 16 Italian regions that allow to define the performances through the LEA levels and the IC elements. The last year available for completeness of the analyzed data and availability of Essential Levels of Assistance evaluation by the Italian Ministry of Health is 2016, therefore the study considers the same year also for the IC elements.

The following table shows the statistical analysis of the input/output variables used by data envelopment analysis model. The results provided in the table are the result of the descriptive statistics, which takes into account the regions considered.

The technologies represent the set of laboratories, image diagnostics, and instrumental diagnostics of health structures. The average of technologies in possession of healthcare facilities in Italy are 8575,938, the region that has the least technology facilities available is Molise, while the one that has the most is Lombardia. Seven of the sixteen regions have several types of equipment above average.

Human resources are represented by doctors and nurses employed by healthcare facilities in each region. The literature highlights how doctors and nurses are the capital with a higher incidence in the treatment process [16, 19, 106]. The average human resources are equal to 20,172.5; the region with the least health professionals available is Molise, while the one with the maximum number of professionals is Lombardia. Nine regions exceed the national average.

The knowledge and skills were deduced from the total number of health training courses provided in each region by the structures accredited by the Ministry of Health of Italy. On average in Italy 1896.5 courses were provided. The region with a lower number of refresher courses provided is Molise, while Lombardia is reconfirmed with greater availability of capital also as regards the skills and knowledge provided. Nine out of sixteen regions exceed the national average of the courses provided.

The perceived quality satisfaction concerning medical and nursing services represents the capital relationship and is equal to a national satisfaction level of 37.761. The evaluation of customer satisfaction is assessed on a scale from zero to one hundred. The region with a lower perception of the quality of the health services provided is Molise, while the one with the highest perception is Emilia Romagna. Nine out of sixteen regions exceed national perceived quality.

The overall evaluation methodology includes a weight system that assigns a reference weight to each indicator and assigns scores with respect to the level reached by the region against national standards. The national level of the Essential Levels of Assistance is equal on average to 182.437; the region with the best score is Veneto while the worst is Campania. Seven regions are below the national average; however, the Ministry of Health considers only two regions out of sixteen (Campania and Calabria) to be non-compliant, confirming a sufficient level of quality provided at the national level for most regions.

Pearson’s correlation index between the variables considered allows us to affirm the absence of correlation and the possibility of excluding a possible relationship between them in future statistical analyses [125, 126] Table 2.

**Table 2:** Matrix of the correlations between variables

Variables	Structural Capital (n° diagnostic tools)	Human capital (n° doctors and nurses)	Human capital (knowledge and skills of workers - n° training courses)	Relational Capital (consumer satisfaction)	Performance (LEA)
Technology Structural Capital (n° diagnostic tools)	1.0000				
Human capital (n° doctors and nurses)	0.3412	1.0000			
Human capital (knowledge and skills of workers - n° training courses)	0.2492	0.9640	1.0000		
Relational Capital (consumer satisfaction)	0.0562	0.8587	0.8454	1.0000	
Performance (LEA)	0.8373	0.3525	0.3040	0.0745	1.0000

## Results

**Fig. 2:**
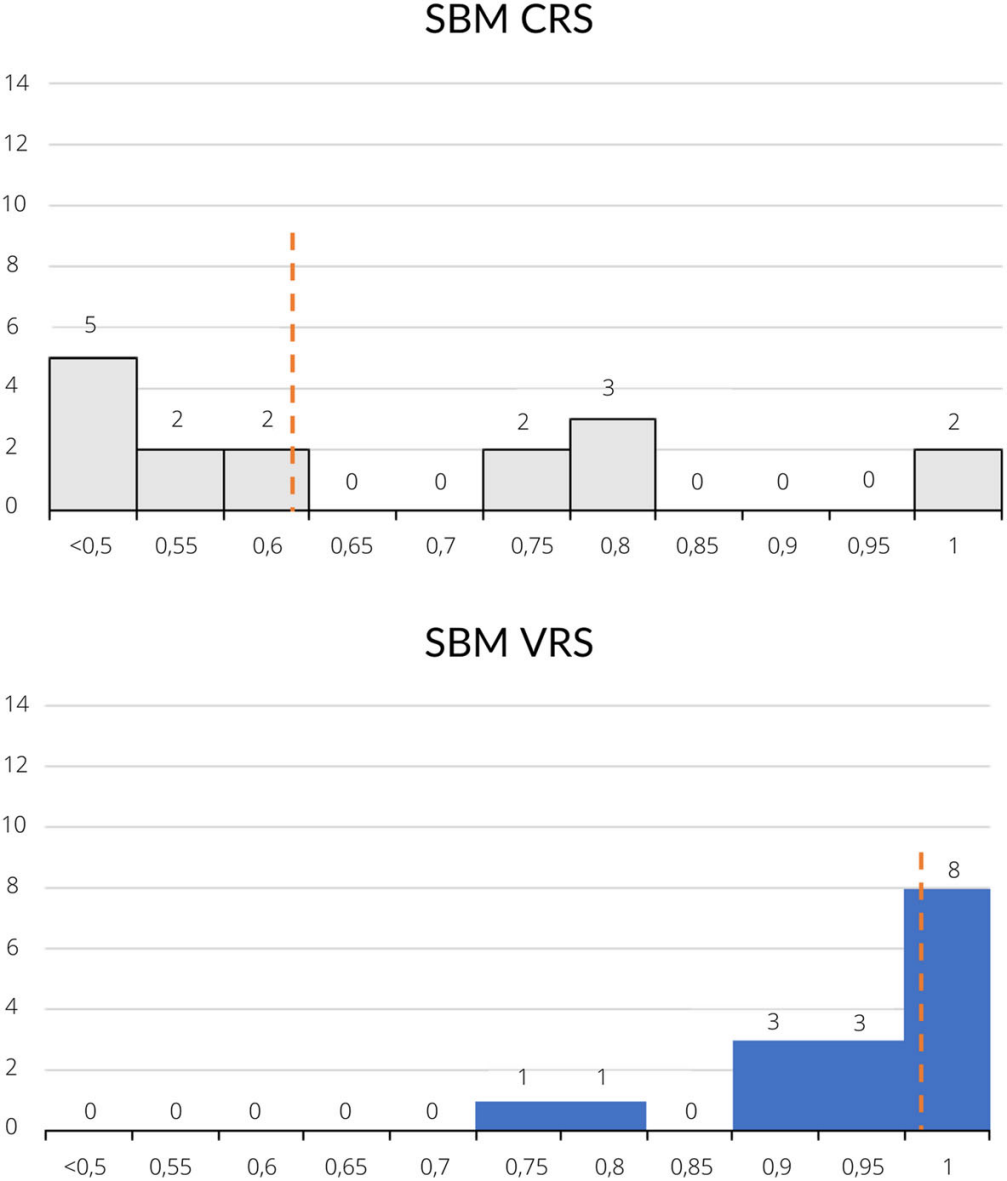
Histogram of the efficiency scores in the SBM-CRS and the SBM-VRS. Source of figure: own production

Table 3 lists the relative efficiency for the 16 regions in 2016 which were obtained from SBM-CRS and SBM-VRS models. In SBM-CRS model, the efficiency score (OTE) ranges from 0.415 to 1.000, with an average of 0.636 (the dotted line in Fig. 2) and a standard deviation of 0.192.

**Table 3:** Efficiencies in the 16 regions in 2016 based on the SBM-CRS and SBM-VRS models

No.	DMU	OTE-CRS	PTE-VRS	SE	RTS
1	Piemonte	0.4812	10.000	0.4812	Decreasing
2	Lombardia	0.4154	0.9474	0.4385	Decreasing
3	Veneto	0.5690	10.000	0.5690	Decreasing
4	Liguria	0.5667	10.000	0.5667	Decreasing
5	Emilia Romagna	0.4152	0.9844	0.4218	Decreasing
6	Toscana	0.5208	10.000	0.5208	Decreasing
7	Umbria	0.5156	10.000	0.5156	Decreasing
8	Marche	0.4903	0.9593	0.5111	Decreasing
9	Lazio	0.7706	0.9857	0.7818	Decreasing
10	Abruzzo	0.4375	0.9481	0.4614	Decreasing
11	Molise	10.000	10.000	10.000	Constant
12	Campania	0.7184	0.7306	0.9833	Decreasing
13	Puglia	10.000	10.000	10.000	Constant
14	Basilicata	0.7726	10.000	0.7726	Decreasing
15	Calabria	0.7395	0.8443	0.8759	Decreasing
16	Sicilia	0.7611	0.9160	0.8309	Decreasing
Descriptive statistics of the efficiency score					
	OTE-CRS		PTE-VRS		SE
Average	0.636		0.957		0.671
Max	1000		1000		1000
Min	0.415		0.731		0.422
St. Dev	0.192		0.074		0.215

The number of efficient regions is just 2, indicating that only 12,5% of the sample are efficient.

The efficiency scores of 68% of regions are less than 0.75. Therefore, the major part of regions are strongly inefficient.

The efficiency score (PTE) ranges from 0.731 to 1.000 in the SBM-VRS model, with an average of 0.957 (the dotted line in Fig. 2) and a standard deviation of 0.074.

The number of efficient regions is 8, indicating that 50% of the sample are efficient.

The efficiency scores of only 6% regions are less than 0.75.

From the analysis of the summary of the return to scale (RTS), it shows that 14 regions are operating under decreasing returns to scale (DRS) and 2 under constant returns to scale (CRS). The DRS occurs if the proportionate increase in all of the inputs results is less than the proportionate increase in its outputs, while in the constant return to scale, units operates if there is an increase in inputs resultant in a proportionate increase in the output levels. This underlines that an important effort is required in improving the IC dimensions to reach efficient levels.

Table 4 lists the results of input and output slack. The remaining 8 inefficient regions have some room to improve in terms of input/ output slack, as listed in Table 4. The results demonstrate that the inefficient regions can improve if the input values are markedly reduced by the gross input improvement and the output values are augmented by the output improvement. It is very important for inefficient regions to discover which input or output factors require modification so that policy makers could identify the major problems of the regional healthcare system based on the results.

**Table 4:** input and output slack for each DMU

		Slack	Slack	Slack	Slack	Slack
No.	DMU	Structural Capital (n° diagnostic tools)	Human capital (n° doctors and nurses)	Human capital (knowledge and skills of workers - n° training courses)	Relational Capital (consumer satisfaction)	LEA
1	Piemonte	0	0	0	0	0
2	Lombardia	10,930.86	14,935.68	2,441.977	1.23	11,002
3	Veneto	0	0	0	0	0
4	Liguria	0	0	0	0	0
5	Emilia Romagna	0	875,559	847,736	1.19	3239
6	Toscana	0	0	0	0	0
7	Umbria	0	0	0	0	0
8	Marche	0	821.3	85.133	0	8155
9	Lazio	2,692.891	1,373.823	3,149.849	0	2592
10	Abruzzo	0	1,210.524	69.014	5.23	10.345
11	Molise	0	0	0	0	0
12	Campania	45.457	5,898.694	1,086.047	0	45.712
13	Puglia	0	0	0	0	0
14	Basilicata	0	0	0	0	0
15	Calabria	0	5,117.41	430.508	0	26.56
16	Sicilia	991.389	2,703.636	295.245	0	14.952

Among the sample, the half (8 regions) is believed to manage intellectual capital more efficiently than the others.

The results of ratio analysis of output/ input values comparing efficient and inefficient regions (as shown in Table 5) indicate that the values of Essential Levels of Assistance and changes of intellectual capital stocks for efficient regions exceeded those for those inefficient. Efficient regions performed considerably better in terms of every ratio of outputs over inputs. Based on the input and output slacks as listed in Table 4, it is suggested that inefficient regions seeking to catch up with the efficients must reduce their employee numbers to achieve an efficient frontier and optimize the number of technological tools.

**Table 5:** Ratio analysis of the output/input values with respect to regional efficiency

	Structural Capital (n° diagnostic tools)	Human capital (n° doctors and nurses)	Human capital (knowledge and skills of workers - n° training courses)	Relational Capital (consumer satisfaction)
Efficient Regions	0.0561	0.0273	0.3970	55.836
Inefficient Regions	0.0283	0.0101	0.1233	51.134
Average	0.0422	0.0187	0.2601	53.485

This situation indicates that policy makers need to be extremely cautious in monitoring the performance and effectiveness of use of technology. Executives should be aware of the efficiency of resource allocation in seeking to obtain competitive advantages in relation to effect of intellectual capital on performance, in terms of Essential Levels of Assistance.

In the second phase, the efficiency score is considered as a dependent variable to study how exogenous variables impact it; in particular, the number of residents and the current expenditure according to the literature could influence health performance. The resources allocated in the system that can be defined through the volumes of expenditure in the income statement in each region could condition the result in terms of performance achieved by each region as regards the fallout of the treatment process [117, 127]. The Italian healthcare system, based on an allocation of resources for each resident citizen [128], could be found on the number of residents that affect the performance of each regional health system [129]. These elements are considered in the analysis conducted in order to verify and confirm the existing theory. All the elements analyzed can influence to help the political decision makers and the public managers during the planning and control planning phases of the correct volume of resources that each region should have in order to reach adequate performance levels. In fact, in healthcare, performance can be assessed as the best level and mix of efficiency, effectiveness and equity with the resources available [130].

The regional efficiency score calculated through a multivariate linear regression is indirectly proportional to the resident population (β= − 0.0002549, standard error 0.000805, *p* = 0.007, and *R*2 = 0.4575) and directly proportional to current expenditure (β= − 0.0001331, standard error 0.000431, *p* = 0.009, and *R*2 = 0.4575). In order to confirm the statistical result obtained through multivariate regression, a Tobit analysis was conducted [131]. The result confirms what has already been stated; there is an indirect relationship between the efficiency score obtained and the population (*p* = 0.004), and a direct relationship between the efficiency score and the expense (*p* = 0.005).

## Discussion

The analysis and investigation are conducted to confirm the trend identified in the literature [29]. It tries to identify the best tool to investigate the balance of IC and the possible impact in terms of non-financial performance by evaluating any relationship on financial factors. Both the critical studies on IC [21] and those that identify the relationship between IC and performance [17] immediately highlight the absence of regional and national studies, very often identifying an identifiable limitation to the single case study. For the first time compared to what has been achieved, evidence related to the country’s entire national system is considered. Italy is an excellent case study for its positioning within European and global healthcare and for specific characteristics that distinguish it and ensure that there are no distortions related to the services provided, being the universal health system and accessible to all with the same procedures. Although they have a different allocation of resources, the structures present on the regional territories are organized and managed uniformly in the regions considered. The data collected and the performance evaluation is uniform and applies the same criteria. Unfortunately, the ICs collected and systematized at a national level are not numerous, although they are sufficient to identify a first survey model and postulate the first hypotheses. Several studies have used the questionnaire to identify relationships, weight, and use of ICs concerning financial and non-financial performance. The analysis performed is innovative and overcomes the limitation linked to the mere creation of theoretical frameworks without empirical evidence of use [3]. The use of data envelopment analysis allows to identify the correct allocation of resources on the basis of the desirable outputs (Essential Levels of Assistance). In data envelopment analysis, potential performance is not calculated based on theoretical criteria but rather is based on comparison with other DMUs. Thus, the addition or removal of DMUs will most likely yield a different set of efficiency scores for the companies. Such additions or deletions could alter the set of companies lying on the efficient frontier. Additionally, as data envelopment analysis calculation is based on surrogate input and output variables, if the study misses some necessary measures (that is, intangible assets), the researchers should verify its reliability and validity. The sample analyzed shows that there is no balance between IC and non-financial performance in most Italian regions; there are only two regions that have effectively placed resources obtaining an efficient level of Essential Levels of Assistance. The model allows us to understand that the lack of technologies in most cases and the excess of human resources do not lead to satisfactory results. The model also allows us to define an evaluation criterion for allocating the ICs in the national and regional contexts. The health administration of each region is autonomous. However, the allocation of resources depends on a series of national criteria and policies based not only on the number of residents but also on the definition of transfers aimed at balancing regional efficiency by eliminating differences. The model is also useful for considering certain factors such as the incidence of the number of residents with respect to the performance obtained and obtainable and the impact that expenditure has on the assessment of non-financial performance. In particular, the analysis highlights that the greater the number of residents, the less quality will be achieved according to the levels defined by the model. This implies that the greater the number of residents, the greater the attention should be given to the ICs made available, and that the greater the expenditure in each region, the greater the achievement of optimal results in terms of performance. Therefore, it is evident that the relationship between the performance indicator obtained by the data envelopment analysis and expenditure shows a consequent effective relationship also between expenditure and IC. Therefore, the new model allows an initial definition of the allocation of resources. The data envelopment analysis surpasses the previous balanced scorecard instrument introduced with the NPM in the healthcare sector. The model was based on a set of measures that allowed strategic perspectives taking into account financial elements, customers, internal business process, learning, and growth. The model allowed for a balance between the short and long term. However, the literature introduced on IC and performance evaluation highlights how some elements have somehow been confused between what are the inputs and the system outcomes. As evidenced by Peng’s study [19], the economic factor is an output, and the allocation of resources is utilitarian for the strategic definition of the budget. Therefore, the financial aspect is not an autonomous input that helps define the levels but a system outcome. Suppose the Balanscored card allows continuous improvement with the definition of a vision and strategy that considers objectives, measures, targets, and initiatives for each element. It does not allow a definition at a regional level where the analysis of all the factors would create a significant increase in costs, giving by an excess of information and microanalysis which have to be conducted. On the other hand, the proposed approach provides a proposal for a tool in line with the needs of PMS that immediately identifies a coherent strategy based on regional health performance [3, 52]. Managers already consider the value of ICs within hospitals, but there are still no studies that provide regional and national policymakers and public managers with guidance on the use of available data. Nevertheless, in Italy, the collection of information is limited and does not take all the ICs. Furthermore, it is impossible to have data updated in real-time, and this significantly limits the perspective of accurate programming. Nonetheless, policymakers currently rely on available data, so the approach adopted can still be considered valid for future planning.

## Conclusions

The NPM theory and the overcoming of previous Performance Measurement System tools within the national and regional contexts for the allocation of resources are addressed in the study in light of the influence that ICs provide on health performance [28]. The increase in the average age and austerity actions on spending leads to the search for sustainable resource allocation tools, and the study is part of the international debate on the correct use of available resources [3, 22]. It enriches the studies that consider the non-financial performance.

This research focuses on the relationship between intellectual capital and performance level of the regional healthcare system in Italy using data envelopment analysis. This study demonstrates that half of the sample has achieved a satisfying level of efficiency. The empirical results also help to identify the benchmark regions which allow public decision making to guide the choice of composition of the resources distributed in each region according to the IC criterion. The research results suggest that intellectual capital, which comprises human capital, relational capital, and structural capital, is one of the main sources of competitive advantage in the healthcare sector. This study argues that intellectual capital is a necessary strategic tool for regional and managerial policy. The emphasis of the IC can help policymakers and executives to implement new initiatives for enhancing regional performance system, considering not only the expenditures aspect. Measuring the efficiency of intellectual capital management will allow regions to understand if they have efficiently managed the public resources available. The analysis model also guarantees greater management capacity in a period of scarcity of available resources with a progressive increase in inhabitants. The study confirms the impact of some exogenous factors, such as the number of residents and current expenditure, on the efficiency score. The current study focuses on one country and should be traced back to standard variables on other countries to confirm what the empirical analysis shows. The different national organizational and systemic contexts linked to health could lead to different results that could require further investigation and analysis. Nevertheless, the approach is innovative and can be re-proposed by further investigating the future of the healthcare sector, which suffers from a lack of empirical attention in light of the expected demographic trend.

The study has several limitations, it does not consider some variables, which according to the literature may have an impact on intellectual capital and consequently on performance, but which at present the availability of data does not allow a precise system analysis, among these the literature identify leadership skills [132] and relational elements of networks between subjects [17, 21] also secondary variables that cold impact on the performance evaluation of the healthcare sector [17].

## Data Availability

Data sharing is not applicable to this article as no new datasets were generated or analysed during the current study. Data used are available on public repository cited in the references section.

## Abbreviations

CRS: Constant returns to scale
DEA: Data envelopment analysis
DMUs: Decision-making units
DRS: Decreasing return to scale
IC: Intellectual capital
LEA: Essential Levels of Assistance (Livelli Essenziali di Assistenza)
NPM: New Public Management
OTE: Overall technical efficiency (determined in SBM-CRS model)
PMS: Performance measurement systems
PTE: Pure technical efficiency (determined in SBM-VRS model)
RTS: Return to scale
SBM-CRS: Slack-Based Model constant returns-to-scale
SBM-VRS: Slack-Based Model Variable return-to-scale
SE: Scale efficiency
