# Correction to: Intellectual capital-based performance improvement: a study in healthcare sector

**DOI:** 10.1186/s12913-021-06127-7

**Published:** 2021-02-22

**Authors:** Simona Alfiero, Valerio Brescia, Fabrizio Bert

**Affiliations:** 1 Department of Management, C.so Unione Sovietica, University of Turin, 218 bis, Torino, Italy; 2 Department of Public Health Sciences, University of Turin, Via Santena 5/bis, Torino, Italy

**Correction to: BMC Health Serv Res 21, 73 (2021)**

**https://doi.org/10.1186/s12913-021-06087-y**

Following the publication of the original article [1], the authors identified an error in Table 3. The correct table is given below.

**Table 3:** Efficiencies in the 16 regions in 2016 based on the SBM-CRS and SBM-VRS models

No.	DMU	OTE-CRS	PTE-VRS	SE	RTS
1	Piemonte	0.4812	1.0000	0.4812	Decreasing
2	Lombardia	0.4154	0.9474	0.4385	Decreasing
3	Veneto	0.5690	1.0000	0.5690	Decreasing
4	Liguria	0.5667	1.0000	0.5667	Decreasing
5	Emilia Romagna	0.4152	0.9844	0.4218	Decreasing
6	Toscana	0.5208	1.0000	0.5208	Decreasing
7	Umbria	0.5156	1.0000	0.5156	Decreasing
8	Marche	0.4903	0.9593	0.5111	Decreasing
9	Lazio	0.7706	0.9857	0.7818	Decreasing
10	Abruzzo	0.4375	0.9481	0.4614	Decreasing
11	Molise	1.0000	1.0000	1.0000	Constant
12	Campania	0.7184	0.7306	0.9833	Decreasing
13	Puglia	1.0000	1.0000	1.0000	Constant
14	Basilicata	0.7726	1.0000	0.7726	Decreasing
15	Calabria	0.7395	0.8443	0.8759	Decreasing
16	Sicilia	0.7611	0.9160	0.8309	Decreasing
Descriptive statistics of the efficiency score					
	OTE-CRS		PTE-VRS		SE
Average	0.636		0.957		0.671
Max	1.0000		1.0000		1.0000
Min	0.415		0.731		0.422
St. Dev	0.192		0.074		0.215

The original article has been corrected.
